# Gene expression alterations in salivary gland epithelia of Sjögren’s syndrome patients are associated with clinical and histopathological manifestations

**DOI:** 10.1038/s41598-021-90569-w

**Published:** 2021-05-27

**Authors:** Ariana Dela Cruz, Vinay Kartha, Andrew Tilston-Lunel, Rongjuan Mi, Taylor L. Reynolds, Michael Mingueneau, Stefano Monti, Janicke L. Jensen, Kathrine Skarstein, Xaralabos Varelas, Maria A. Kukuruzinska

**Affiliations:** 1 Department of Translational Dental Medicine, Boston University School of Dental Medicine, Boston, USA; 2 Department of Medicine, Boston University School of Medicine, Boston, USA; 3 Department of Biochemistry, Boston University School of Medicine, Boston, USA; 4 Immunology Research, Biogen Idec, Cambridge, MA USA; 5 Faculty of Dentistry, University of Oslo, Oslo, Norway; 6 Department of Clinical Medicine, University of Bergen, Bergen, Norway

**Keywords:** Autoimmune diseases, Immunopathogenesis, Biomarkers, Transcriptomics, Salivary gland diseases

## Abstract

Sjögren’s syndrome (SS) is a complex autoimmune disease associated with lymphocytic infiltration and secretory dysfunction of salivary and lacrimal glands. Although the etiology of SS remains unclear, evidence suggests that epithelial damage of the glands elicits immune and fibrotic responses in SS. To define molecular changes underlying epithelial tissue damage in SS, we laser capture microdissected (LCM) labial salivary gland epithelia from 8 SS and 8 non-SS controls for analysis by RNA sequencing (RNAseq). Computational interrogation of gene expression signatures revealed that, in addition to a division of SS and non-SS samples, there was a potential intermediate state overlapping clustering of SS and non-SS samples. Differential expression analysis uncovered signaling events likely associated with distinct SS pathogenesis. Notable signals included the enrichment of IFN-γ and JAK/STAT-regulated genes, and the induction of genes encoding secreted factors, such as LTF, BMP3, and MMP7, implicated in immune responses, matrix remodeling and tissue destruction. Identification of gene expression signatures of salivary epithelia associated with mixed clinical and histopathological characteristics suggests that SS pathology may be defined by distinct molecular subtypes. We conclude that gene expression changes arising in the damaged salivary epithelia may offer novel insights into the signals contributing to SS development and progression.

## Introduction

Sjögren's syndrome (SS) is a debilitating complex autoimmune disease presenting as exocrinopathy of salivary and lacrimal glands with frequent systemic extraglandular manifestations and an increased risk of non-Hodgkin’s lymphoma^1–6^. Damage to salivary and lacrimal glands from SS impairs their ability to produce saliva and tears, resulting in xerostomia and xerophthalmia. SS is a multifactorial disease that comprises various combinations of dry eyes, dry mouth, reduced tear and saliva secretion, presence of autoantibodies, focal lymphocytic infiltration of salivary glands, fatigue, vasculitis, joint and muscle pain, as well as peripheral nervous system dysfunction^4,7^. Although to date, the origin of SS remains unknown, the prevailing model for SS has been that loss of secretory function is secondary to lymphocytic infiltrates.

Since defects in the immune system have long been considered to be the cause of SS, the majority of studies and therapeutic approaches have focused on aberrant immune responses^8–10^. Analysis of genomic and epigenomic changes in SS has provided new insight into the understanding of disease pathogenesis^9,11–13^. Gene expression microarray studies on labial salivary glands and peripheral blood detected dysregulation of type 1 interferon (IFN)-inducible genes^14^. Additional upregulated genes involved in antigen presentation, lymphocyte development and activation, as well as interferon-induced chemokines were also identified. Interestingly, IFN-γ-inducible genes, such as Signal transducer and activator of transcription (STAT) family members STAT1 and STAT3, were also shown to be highly expressed in primary SS (pSS)^15^. Furthermore, IFN-mediated innate immune mechanisms have been implicated in the pathogenesis of pSS, where specifically 23 genes known to play a role in IFN signaling were identified^16^. Two GWAS studies have been performed in pSS and among the SS-associated non-HLA genes they identified STAT4 and IRF5 encoding transcription factors, BLK coding for B cell kinase as well as genes encoding the IL-12A cytokine. Genes involved in NF-κB signaling and CXCR5 chemokine production were also shown to be upregulated^9,17^. Collectively, these studies provided a strong support for the role of innate immunity, as well as adaptive immune mechanisms, in the pathogenesis of pSS. Despite these advances, treatment strategies for SS disease are almost inexistent, and molecular mechanisms that drive disease onset are poorly understood.

One consistent feature of human autoimmunity is the finding that there is an initial phase in which patients have many of the serological abnormalities of autoimmune disease before they develop clinical pathology^18^. In this respect, the immunobiology of the glandular epithelial cells within the target tissue likely plays a critical role in initiating events in SS. Indeed, accumulating evidence suggests that impaired glandular secretion develops independent of lymphocytic infiltration, and defective secretion precedes the immune response during SS onset, suggesting a role for the epithelium in the etiology of SS^2^. For example, aberrantly high expression of BMP growth factors, such as BMP6, has been implicated in the induction of salivary and lacrimal gland dysfunction independent of the autoantibodies and immune infiltration associated with SS^19^. Recent studies, including our own, have also shown that the structural integrity of the epithelium of SS patients is lost, and that these defects are associated with aberrant apical-basal polarity^20–25^. Aberrant polarity can arise in various ways, including environmental toxins, viral infection, physical damage, and unbalanced hormone levels, and therefore it may be an early event that triggers disease states, including SS. Since structural changes of the epithelium of salivary glands may drive inflammation, the aim of this study was to elucidate molecular details of how epithelial structural dysfunction contributes to SS. We used laser capture microdissection of glandular epithelial regions (acini and ducts) from resected labial salivary glands of patients fulfilling or not fulfilling SS criteria (8 SS and 8 non-SS controls) and processed them for global transcriptomes using bulk RNAseq analysis. Our studies provide evidence that damage to salivary gland epithelia is associated with changes in gene expression signatures that provide insights into deregulated pathways and cellular processes which may underlie predisposition to, and early onset of, SS. Collectively, these findings provide support for the theory that alterations in gene expression arising in the salivary epithelia contribute to the etiology of SS. We postulate that the molecular sub-groups reflect varying stages of disease predisposition and that they may offer novel insights into the signals contributing to the progression of SS.

## Results

### Clinical characteristics of patients

Sixteen samples of labial salivary glands were obtained from female patients who presented with sicca symptoms to the Department of Otolayngology/Head and Neck Surgery at Haukeland University Hospital, Bergen. Patients were assessed for primary Sjögren’s Syndrome (pSS) based on the American-European Classification Criteria^26^, where diagnosis of pSS required focal lymphocytic sialadenitis score (FS) of ≥ 1 or presence of anti-SSA/Ro or anti-SSB/La autoantibodies, as well as ocular symptoms of dry eyes, oral symptoms of dry mouth, positive Schirmer’s test, and reduced unstimulated salivary flow ≤ 1.5 mL in 15 min (at least four positive tests including symptoms, or three objective tests). We categorized samples into two clinically distinct groups, patients diagnosed with SS (named SS, n = 8) or without SS (named non-SS, n = 8). Clinical characteristics of patients are shown in Table 1. The patient mean age was 53 ± 12.4 years, with three patients in the SS group presenting with anti-SSA/Ro and ANA antibodies, of which also two were positive for anti-SSB/La antibodies. All patients reported dry eyes and the majority reported dry mouth. Three SS patients had FS of 1, three had a FS of 2, with one patient presenting with FS of 3 and one with FS of 4. Interestingly, FS of either 1 or 4 were associated with positivity for all anti-SSA/Ro, anti-SSB/La and ANA, suggesting that these may be independent variables in our sample group. Likewise, there was no association between germinal centers (GCs) and autoantibody positivity. Furthermore, two of the non-SS patients had a negligible salivary flow rate, and there was little correlation between salivary flow rate and auto-antibody positivity in SS patients.

**Table 1:** Clinical characteristics of patients whose salivary gland biopsies were analyzed by RNAseq.

Clinical classification	Patient	Age	Gender	Anti-SSA/Ro	Anti-SSB/La	ANA	Schirmer test	Saliva secretion (ml/15 min)	Dry mouth	Dry eyes	FS	Germinal Centers (CD21^+^)
Non-SS	Non-SS1	45	F	−	−	−	+	0.5	+	+	−	−
	Non-SS2	53	F	−	−	−	−	0.0	+	+	−	−
	Non-SS3	51	F	−	−	−	−	0.0	+	+	−	−
	Non-SS4	51	F	−	−	−	+	3.2	+	+	−	−
	Non-SS5	34	F	−	−	−	+	1.0	+	+	−	−
	Non-SS6	59	F	−	−	−	+	2.6	+	+	−	−
	Non-SS7	32	F	−	−	−	+	4.0	−	+	−	−
	Non-SS8	40	F	−	−	−	+	2.1	+	+	−	−
SS	SS1	62	F	+	+	+	−	1.7	+	+	1	−
	SS2	74	F	−	−	−	+	0.9	+	+	2	+
	SS3	51	F	−	−	−	+	1.7	+	+	1	−
	SS4	56	F	−	−	−	+	2.8	+	+	1	−
	SS5	53	F	−	−	−	+	1.8	+	+	2	+
	SS6	79	F	+	+	+	NT	1.0	+	+	4	+
	SS7	55	F	−	−	−	+	4.4	+	+	2	+
	SS8	52	F	+	−	+	+	6.6	−	+	3	+

*ANA* antinuclear antibodies; Saliva secretion (unstimulated flow); normal flow > 1.5 ml/15 min; Schirmer test positive when reduced tear secretion (objective test); Dry mouth and dry eyes, subjective symptoms from the patient, *FS* Focus Score, *NT* not determined. Germinal center-like structures may result from the deregulated proliferation and infiltration of lymphocytes in glandular tissue.

**Figure 1:**
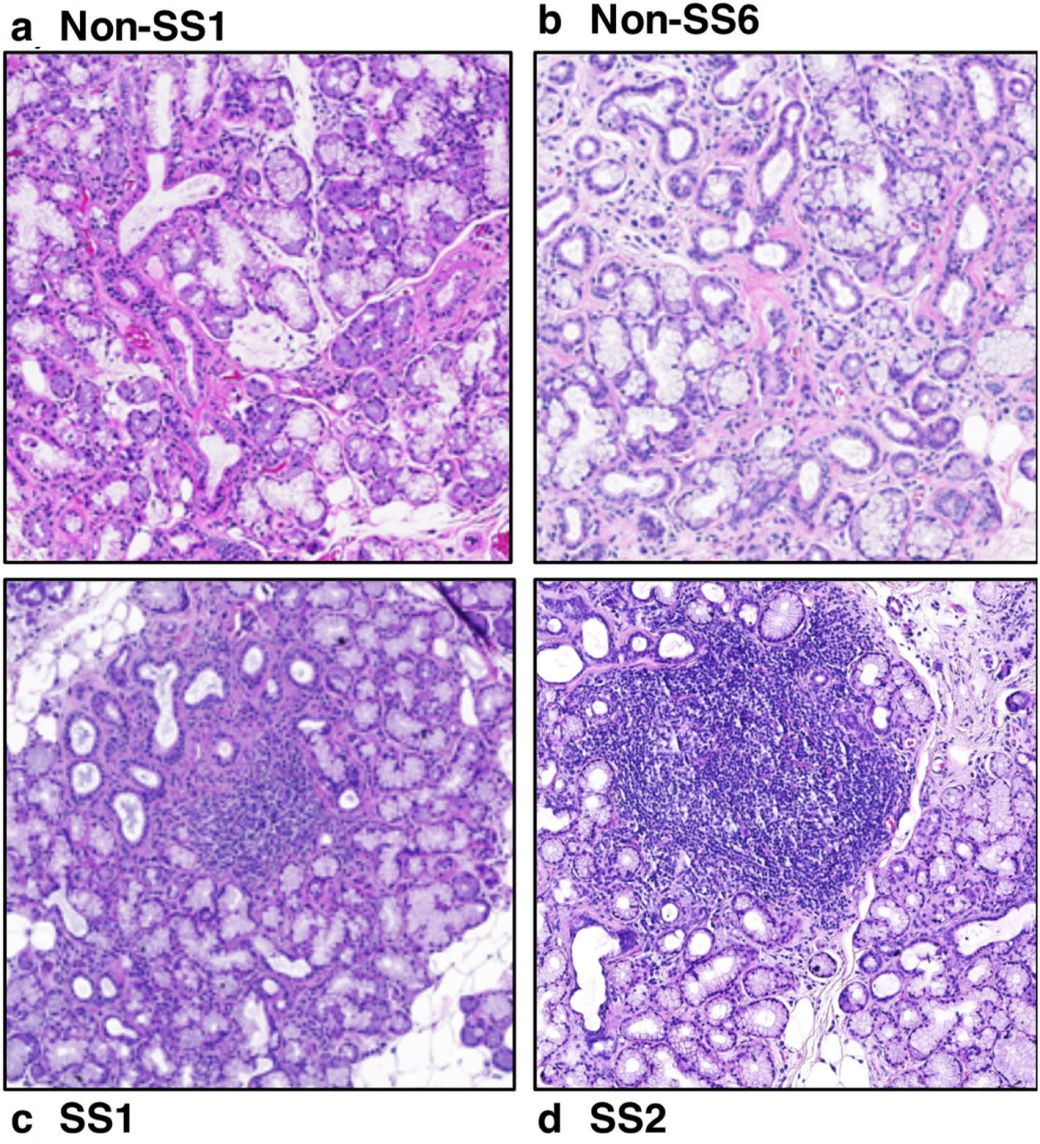
Minor salivary glands biopsies. H&E staining of minor salivary gland biopsies from: (**a**) Non-SS control showing normal appearing salivary gland tissue with few lymphocytic cells, (**b**) Non-SS control with scattered lymphocytes and some interstitial fibrosis, (**c**) SS patient with FS = 1 showing a focal lymphocytic infiltrate and some acinar atrophy and (**d**) SS patient with FS = 2 with a germinal center within a large lymphocytic focal infiltrate.

Histopathological evaluation of H&E sections of biopsies from non-SS and SS patients revealed that SS samples were characterized by acinar atrophy, ductal dilation, detectable fat infiltration, and lymphocytic aggregates with FS ≥ 1, whereas non-SS tissues were generally normal in appearance with some acinar atrophy, ductal dilation, and scattered inflammation (Fig. 1). From analysis of clinical presentation, we observed that there was no correlation between SS and non-SS groups for the parameters of positive Schirmer’s test, saliva secretion, dry mouth or dry eyes. However, the presence of anti-SSA/Ro, anti-SSB/La, ANA autoantibodies, FS ≥ 1, and the presence of CD21+ GCs were only observed in the SS group. When assessed for age, the SS group was significantly older than the non-SS group (60.2 ± 10.7 years vs 45.6 ± 9.6 years respectively; p = 0.012).

### Subclustering of epithelial-derived RNA-seq data reveals potential SS subgroups

**Figure 2:**
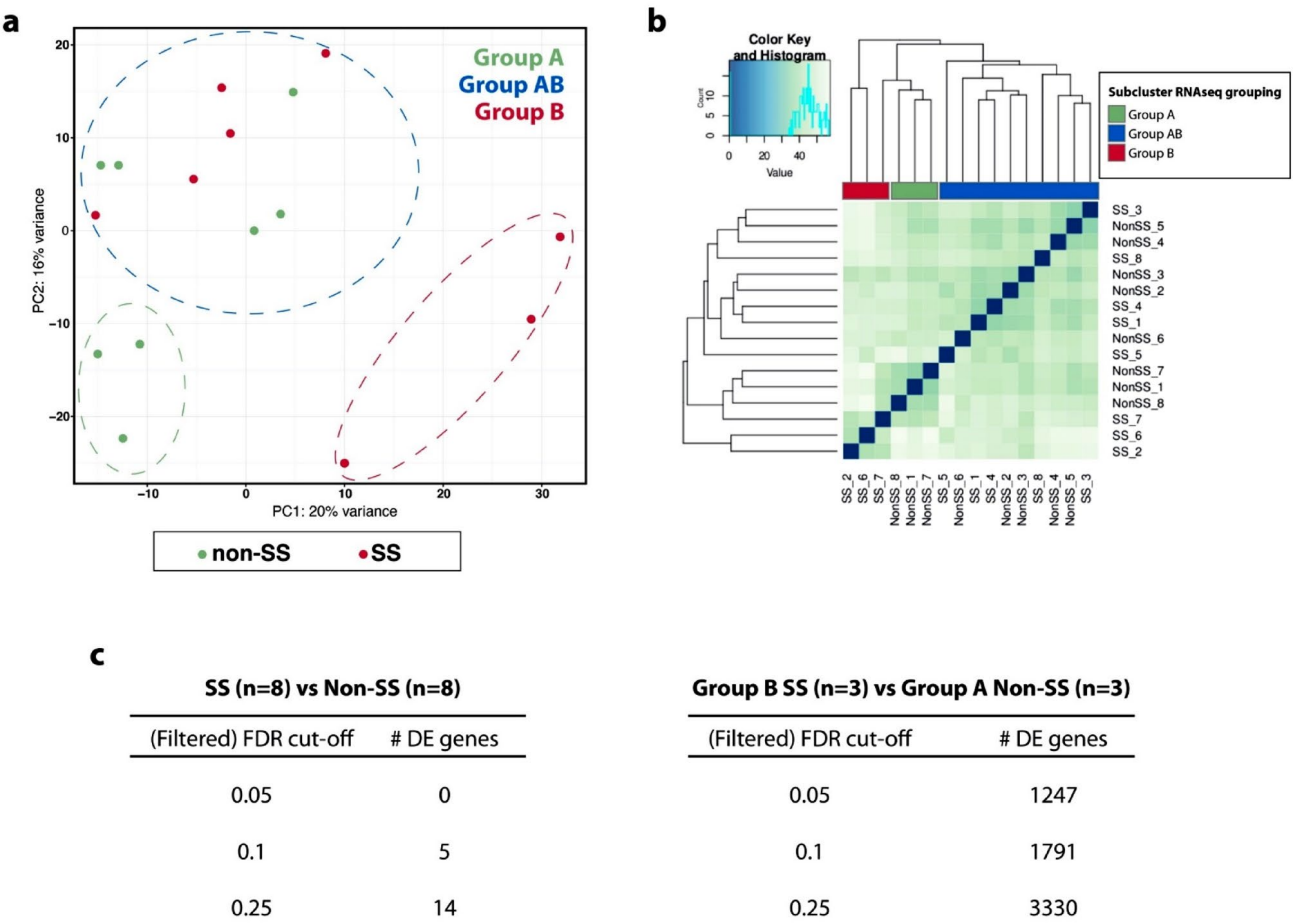
RNAseq of LCM isolated epithelia from 8 non-SS and 8 SS patients. (**a**) PCA of total gene expression data reveals segregation between non-SS (Group A) and SS (Group B) samples and similarities between non-SS and SS (Group AB), identifying a new sub-group with mixed clinical features but the same transcriptional profile. Groups were defined as: Group A (non-SS, n = 3), Group AB (mixed non-SS/SS, n = 10), and Group B (SS, n = 3). (**b**) Sample-sample distance heat map further clusters the sample set. (**c**) Comparison of outputs of the number differentially expressed (DE) genes at different filtered FDR cut-off levels. When the biopsies were analyzed for DE genes by RNAseq cluster, a greater number of DE genes were observed.

To gain insight into the molecular characteristics of two groups with sicca symptoms, eight non-SS and eight SS, all samples were subjected to laser capture microdissection (LCM) of epithelial regions followed by RNAseq analysis to identify biomarkers restricted to the epithelial compartment of the labial salivary glands. We evaluated the entire set by principal component analysis (PCA) selected by the expression level of all differentially expressed (DE) genes. It was evident that the SS and non-SS samples did not entirely cluster together and rather three distinct transcriptomic subclusters were observed: a group containing non-SS samples (named Group A, n = 3), a group containing SS (named Group B, n = 3), and a group containing samples from both groups (named Group AB, n = 10) (Fig. 2a,b). The position of these groupings demonstrates different features of RNA expression in the epithelial tissues, suggesting that transcriptomic profiling of patient samples may characterize important molecular events in SS pathogenesis compared to grouping by clinical characteristics alone.

When assessed for age, Group B (69.3 ± 12.7 years) was significantly older than both the Group AB (52.2 ± 7.4 years; p = 0.020) and Group A (39.0 ± 6.6 years respectively; p = 0.002), whereas there was no significant age difference between Group A and Group AB. Clinical findings for the RNA-Seq groupings revealed that all samples in Group B had GCs with FS of ≥ 2. One of the patients in this group developed lymphoma during the course of this study. In the mixed Group AB, two of the five SS patients also had GCs and lower mean FS. There was no correlation observed for RNAseq grouping for the presence of anti-SSA/Ro, anti-SSB/La and ANA autoantibodies, salivary flow and Schirmer test. Likewise, no specific pattern regarding Group A patients was detected, except that all presented with positive Schirmer tests and a subjective feeling of dry eyes.

### Differential expression testing of SS subgroups

Genome-wide differential expression testing was performed using DESeq2 for multiple group comparisons. First, we tested the clinical grouping of SS against non-SS. Second, we compared the RNAseq grouping of Group B against Group A samples to determine if there were differences in gene expression between clinical diagnosis of Sjögren’s and principal component analysis (PCA) for clustering. Third, the RNAseq Group A and Group B groups were compared to the Group AB.

RNAseq analysis of the epithelial regions for the groupings clinically diagnosed with SS showed no significantly altered differentially expressed (DE) genes compared to the non-SS group out of 25,444 total genes analyzed (Fig. 2c). However, when analyzed according to PCA clustering, there were 1247 DE genes between Group B and Group A at a FDR cut-off of 0.05 (Fig. 2c), further demonstrating a benefit of clustering the samples according to RNAseq data. Table 2 summarizes the top 10 DE upregulated and downregulated genes for both clinical Sjögren’s (SS vs non-SS) and RNAseq (Group B vs Group A vs Group AB) groupings.

**Table 2:** Lists of differentially expressed genes based on RNAseq.

Gene symbol	Description	Fold change (log2)	Adjusted p value
**(a) List of top 10 genes upregulated in SS (n = 8) than non-SS (n = 8)**			
LTF	Lactotransferrin	0.980	0.099
REC8	REC8 meiotic recombination protein	0.947	0.099
HCP5	HLA complex P5 (non-protein coding)	0.935	0.099
SMPX	Small muscle protein, X-linked	0.908	NA
IL15	Interleukin 15	0.882	NA
CA13	Carbonic anhydrase XIII	0.816	0.099
SNORD116-6	Small nucleolar RNA, C/D box 116-6	0.806	0.272
APOL1	Apolipoprotein L, 1	0.802	0.197
HLA-F	Major histocompatibility complex, class I, F	0.793	0.256
CD8A	CD8a molecule	0.792	NA
**(b) List of top 10 genes downregulated in SS (n = 8) than non-SS (n = 8)**			
CA8	Carbonic anhydrase VIII	− 0.762	0.275
CABS1	Calcium-binding protein, spermatid-specific 1	− 0.757	0.275
FDPSP5	Farnesyl diphosphate synthase pseudogene 5	− 0.730	NA
SCD	Stearoyl-CoA desaturase (delta-9-desaturase)	− 0.729	0.275
SCDP1	Stearoyl-CoA desaturase (delta-9-desaturase) pseudogene 1	− 0.725	NA
LINC01207	Long intergenic non-protein coding RNA 1207	− 0.718	0.326
RSL24D1P1	Ribosomal L24 domain containing 1 pseudogene 1	− 0.716	NA
VSIG10L	V-set and immunoglobulin domain containing 10 like	− 0.669	0.348
SEMA3D	Sema domain, immunoglobulin domain (Ig), short basic domain, secreted, (semaphorin) 3D	− 0.668	NA
UNC5A	Unc-5 homolog A	− 0.659	0.408
**(c) List of top 10 genes after RNAseq subclustering that are upregulated in Group B (n = 3) than Group A (n = 3)**			
MMP7	Matrix metallopeptidase 7	2.522	7.36 × 10^–8^
LINC00639	Long intergenic non-protein coding RNA 639	2.297	2.96 × 10^–6^
KCNB1	Potassium channel, voltage gated Shab related subfamily B, member 1	2.274	3.30 × 10^–7^
LINC00284	Long intergenic non-protein coding RNA 284	2.273	3.61 × 10^–6^
CLU	Clusterin	2.223	2.73 × 10^–9^
RARRES1	Retinoic acid receptor responder (tazarotene induced) 1	2.125	2.70 × 10^–6^
C19orf68	Chromosome 19 open reading frame 68	2.119	4.17 × 10^–6^
SH3D21	SH3 domain containing 21	2.062	5.81 × 10^–6^
BMP3	Bone morphogenetic protein 3	2.057	6.22 × 10^–6^
SLC38A3	Solute carrier family 38, member 3	2.022	NA
**(d) List of top 10 genes after RNAseq subclustering that are downregulated in Group B (n = 3) than Group A (n = 3)**			
CALML5	Calmodulin-like 5	− 2.407	4.46 × 10^–17^
VSIG10L	V-set and immunoglobulin domain containing 10 like	− 2.375	9.37 × 10^–15^
HPSE	Heparanase	− 2.152	1.52 × 10^–6^
CABS1	Calcium-binding protein, spermatid-specific 1	− 2.081	7.43 × 10^–6^
MUC7	Mucin 7, secreted	− 2.008	7.88 × 10^–8^
FGF12	Fibroblast growth factor 12	− 1.946	1.28 × 10^–7^
TESC	Tescalcin	− 1.911	1.41 × 10^–6^
DNASE2B	Deoxyribonuclease II beta	− 1.898	0.000155
SCD	Stearoyl-CoA desaturase (delta-9-desaturase)	− 1.863	3.99 × 10^–6^
FADS2	Fatty acid desaturase 2	− 1.853	1.54 × 10^–5^

Inter-group analysis with the Group AB subcluster revealed the following comparisons: 767 DE genes compared to the Group A (FDR cut-off = 0.05) and 1480 DE genes compared to Group B (FDR cut-off = 0.05). While strictly not statistically meaningful, the FDR-value ranking was used to define signatures to be annotated by pathway enrichment analysis.

### Pathway analysis identifies epithelial signals associated with SS

**Figure 3:**
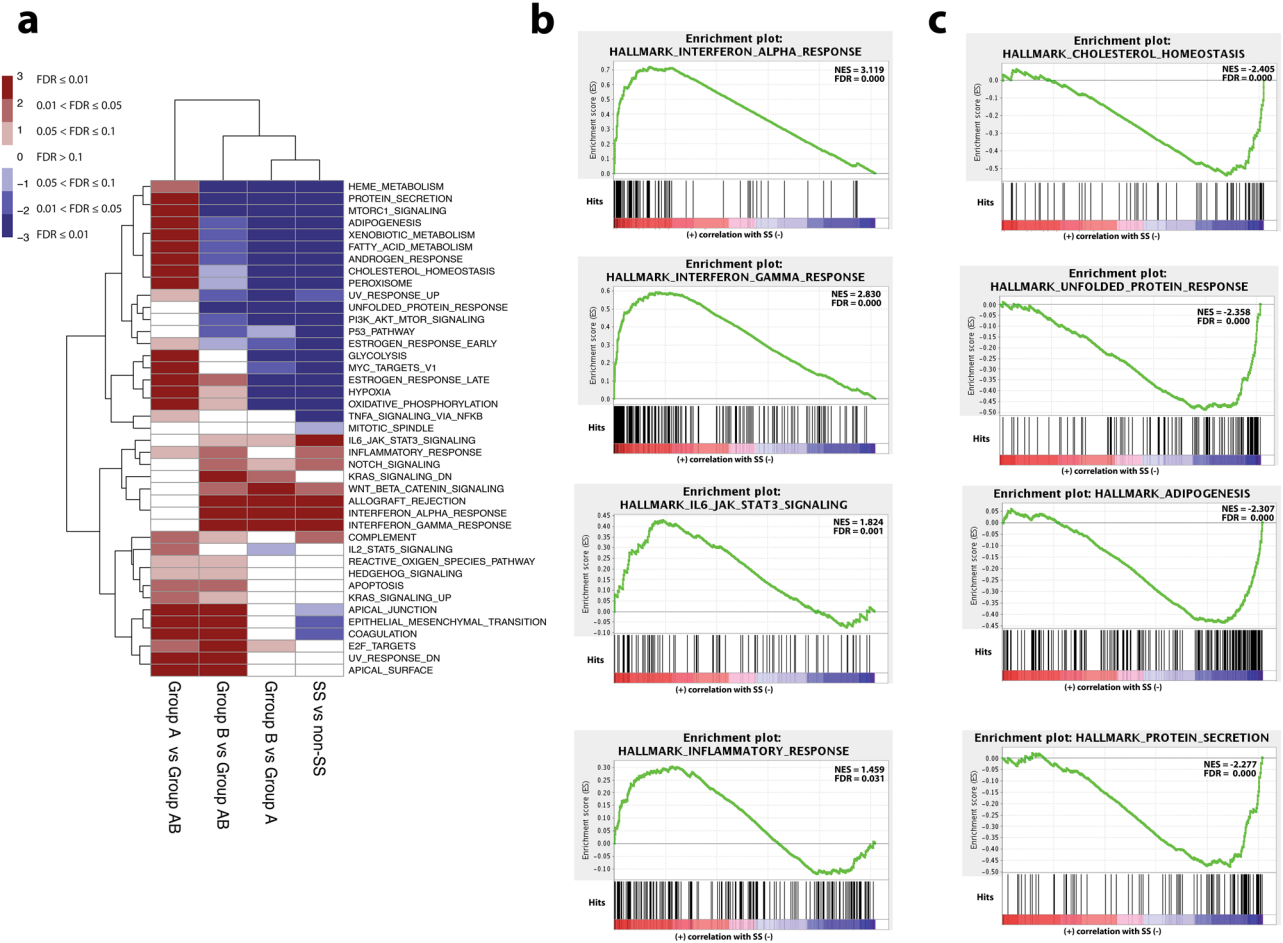
Hallmark gene set analyses from the entire RNAseq dataset. (**a**) Heatmap of hallmark gene sets that are significantly up (red) or downregulated (blue) in the respective indicated pathology. (**b**) Gene set enrichment analysis (GSEA) of hallmark gene sets that are significantly enriched in SS (n = 8) vs non-SS (n = 8). (**c**) GSEA of hallmark gene sets that are significantly enriched in non-SS (n = 8) vs SS (n = 8). *NES *nominal enrichment score, *FDR *false discovery rate. A gene set shows significant enrichment at a FDR < 0.25.

Gene set enrichment analysis (GSEA) was conducted for each of the groups, and hallmark gene sets were evaluated (Fig. 3a). Comparison of SS vs non-SS groupings revealed 8 signaling pathways that were statistically upregulated and 27 pathways downregulated in the SS group (Supplementary Table S1). Notably, immunological and autoimmune disease pathways were enriched in the SS group while cholesterol and protein-related metabolic pathways were downregulated (Fig. 3b,c). Interferon response genes were upregulated in both the clinical SS and RNAseq (Group B) groupings compared to non-SS and Group A samples, respectively, suggesting conservation of relevant processes reflected in RNA profiling. Further hallmark GSEA of comparisons between the subcluster groupings revealed 9 upregulated and 23 downregulated pathways for Group B vs Group A, 21 upregulated and 13 downregulated pathways for Group B vs Group AB, and 30 upregulated and 1 downregulated pathway for Group A vs Group AB (Fig. 3a).

Compared to Group AB, both Group A and Group B showed upregulated pathways in genes encoding components of the apical junction complex and apical surface of epithelial cells, epithelial–mesenchymal transition and coagulation, all indicative of changes in epithelial cell polarity. Significantly, the Group AB showed intermediary expression of genes involved in protein secretion and those activated through the mTORC1 complex as enrichment of genes in these pathways was low in Group B and high in Group A.

While the RNAseq data generated from the epithelia of non-SS and SS salivary glands showed complexity in gene signatures among patient samples, a closer comparison revealed genes that encode secreted proteins that may contribute to the disease. These included genes encoding lactotransferrin (LTF), matrix metalloproteinase 7 (MMP7), bone morphogenetic protein 3 (BMP3), interferon-inducible proteoglycan testican-2 (SPOCK2) as well as cytokines IL-15, IL-19 and BAFF (TNFSF13B). Both BAFF and IL-15 have been shown to be associated with B cell development, physiology and malignancy^13,27^. Furthermore, SPOCK2 was also identified as a downstream target of lncRNA-dependent miRNAs involved in the modulation of ECM genes in SS^13^.

### RNAscope analysis reveals increased LTF, MMP7, and BMP3 expression in Sjögren’s labial salivary gland epithelia

Among the top genes whose transcriptional expression was increased in the RNAseq analysis of the SS grouping were LTF and in Group B were MMP7 and BMP3. LTF is a glycoprotein found in exocrine fluids, such as milk, saliva, tears, and nasal secretions, as well as in secondary granules of polymorphonuclear leukocytes (PMNs) and was previously shown to be upregulated on the protein level in saliva of SS patients^28,29^. MMP7 belongs to a family of ubiquitous proteolytic enzymes, the MMPs, and has been shown to be elevated in corneal specimens of pSS patients^30^. BMP3 is a member of the TGF-β superfamily of proteins, associated with inflammation in rheumatoid arthritis patients with concomitant inhibition of bone repair^31^.

**Figure 4:**
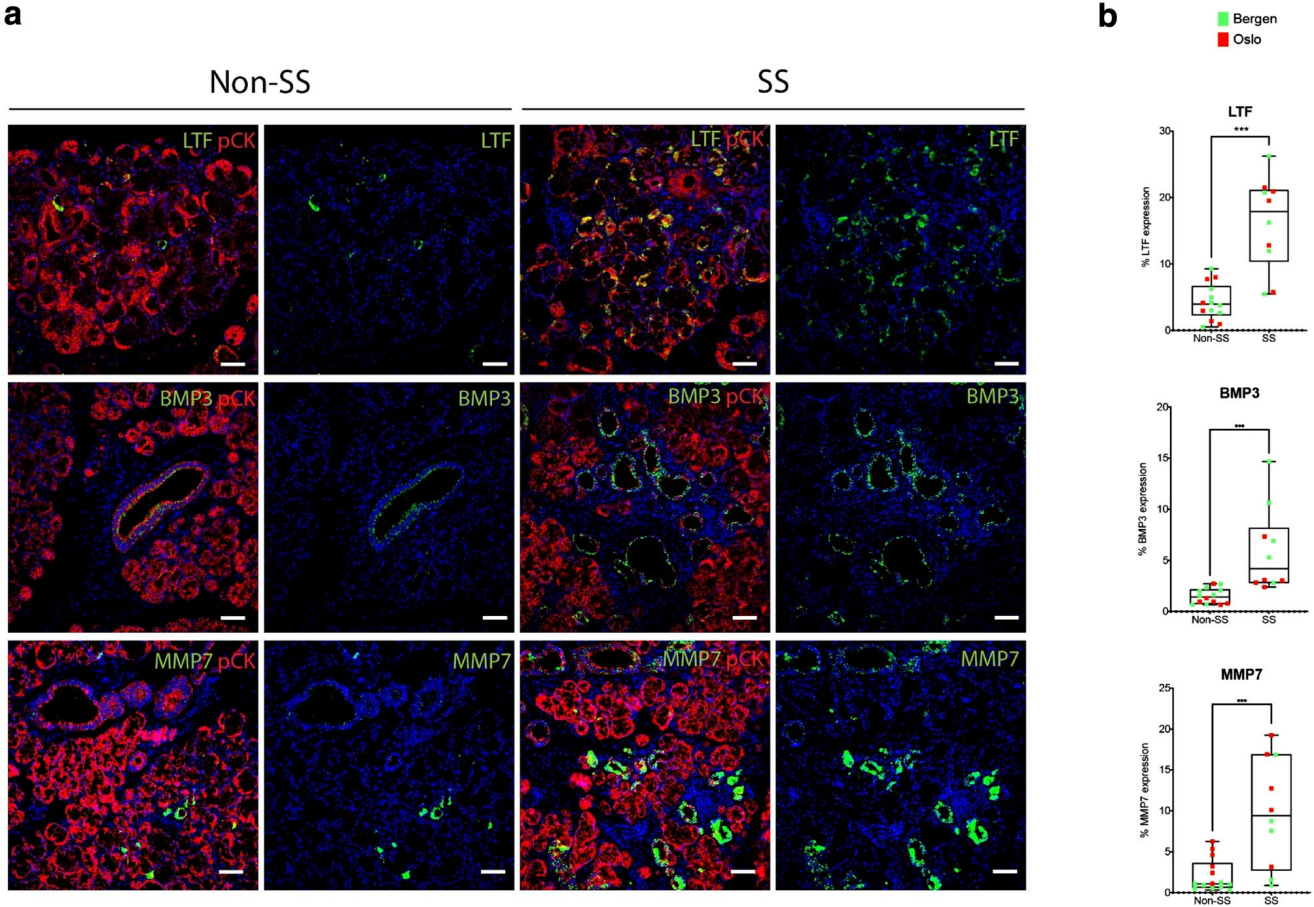
RNAscope validation of gene expression changes identified from RNAseq of SS epithelial tissues. (**a**) SS vs non-SS biopsies were probed for lactotransferrin (LTF), bone morphogenetic protein 3 (BMP3), and matrix metalloproteinase 7 (MMP7) RNA (green) by in situ RNAscope and counterstained with pan-cytokeratin to mark epithelium (pCK, red). Scale bars = 20 µm. (**b**) LTF, BMP3, and MMP7 RNAs were quantified in the respective pCK-positive tissues from patients in the Bergen (green) and Oslo (red) cohorts, revealing increased epithelial expression of LTF, BMP3, and MMP7 in SS tissues. The average % LTF, BMP3, and MMP7 area per patient sample in epithelial regions is shown. Data points represent each patient from Bergen and Oslo cohorts per condition of non-SS or SS. Each data point denotes the average of a range of 2000–4000 epithelial cells scored across multiple tissue fields from each patient sample (***p < 0.001 compared to non-SS group determined with unpaired *t* tests).

In order to validate the increased LTF, MMP7, and BMP3 expression identified in our RNAseq data, we performed independent RNAscope analyses for expression of these genes. This in situ hybridization approach is based on the amplification of target-specific signals but not non-specific background hybridization. Samples were counterstained with pan-cytokeratin in order to restrict analysis of RNA to epithelial regions and analyzed using the CellProfiler image analysis software^32–34^ (http://www.cellprofiler.org). To increase our sample size, we included an additional group of labial salivary gland samples obtained from the University of Oslo. Figure 4 illustrates combined RNAscope analysis of LTF, MMP7 and BMP3 expression from the Bergen samples described in Table 1 and Oslo samples described in Table 3. We note that the age of SS patients in the Oslo cohort was not significantly different than the non-SS group (51.0 ± 19.1 years vs 44.5 ± 13.1 years respectively) although the new Oslo cohort included SS samples from two male patients and the SS groups were mostly positive for autoantibodies (Table 3). Quantitation of the RNA showed significant increases in the epithelial percentage of LTF, MMP3, and BMP3 expression in the SS group compared to the non-SS group (Fig. 4a,b). The observed increases in LTF, MMP3 and BMP3 were not driven by age as there was no significant correlation between patient age and expression of either gene (Fig. 4, “Methods”, “Statistical analysis”). These results correlate with our RNAseq findings of increased epithelial expression of LTF, MMP7, and BMP3 in SS.

**Table 3:** Additional patient samples used for RNAScope (Oslo cohort).

Clinical classification	Patient	Age	Gender	Anti-SSA/Ro	Anti-SSB/La	ANA	Schirmer test	Saliva secretion	Dry mouth	Dry eyes	FS	Germinal centers (CD21^+^)
Non-SS	Non-SSa	42	F	−	−	−	+	**−**	+	+	−	−
	Non-SSb	47	F	−	−	−	+	**−**	+	+	−	−
	Non-SSc	64	F	−	−	−	−	+	+	+	−	−
	Non-SSd	25	F	−	−	−	+	−	+	+	−	−
	Non-SSe	51	F	−	−	−	+	+	+	+	−	−
	Non-SSf	38	F	−	−	−	+	−	+	+	−	−
SS	SSa	49	F	+	+	+	+	+	+	+	1	−
	SSb	30	M	+	+	+	+	+	+	+	1	−
	SSc	38	F	−	−	−	+	+	+	+	2	+
	SSd	79	M	+	+	+	+	+	−	+	1	−
	SSe	59	F	+	−	+	+	+	+	+	1	−

*ANA* antinuclear antibodies; Saliva secretion (unstimulated flow); Schirmer test positive when reduced tear secretion (objective test); Dry mouth and dry eyes, subjective symptoms from the patient, *FS* Focus Score.

## Discussion

Sjögren’s syndrome (SS) is a debilitating autoimmune disease with poorly understood etiology and limited biomarkers available for early detection and prognosis. In this study, we generated and annotated gene expression signatures of labial salivary gland epithelia from human SS and non-SS patients using RNAseq and computational analyses with a goal of obtaining new insights into molecular changes associated with this disease. Our studies provide evidence that damage to salivary gland epithelia is associated with changes in gene expression signatures that provide insights into deregulated pathways and cellular processes which may underlie predisposition to and early onset of SS. We postulate that the molecular sub-groups identified in our studies reflect varying stages of disease predisposition and that they may offer novel insights into the signals contributing to the development of SS.

PCA analysis showed a clear segregation of gene signatures between some non-SS and SS patients (Fig. 2). In addition, it revealed a novel subgroup of gene signatures derived from both non-SS and SS tissues that may reflect a mixed patient population. Interestingly, however, analyses of clinicopathological features of salivary epithelia from SS and non-SS patient cohorts did not reveal any consistent association with GCs and focus scores; indeed, there were no substantial differences among these patient groups in autoantibody positivity, saliva secretion, tear secretion, and subjective feeling of dry eyes or dry mouth. Our identification of a mixed gene expression signature subgroup (Group AB) sheds novel insights into molecular changes in labial salivary glands that may reflect early disease in individuals whose symptoms do not meet the criteria for SS diagnosis.

Better understanding of the molecular features that underlie different subsets of patients is essential for disease diagnostics, prognosis, and effective treatment interventions. The focal infiltration of the salivary gland provides the basis for the focus score used to describe the pathological changes of the salivary glands in the currently used classification criteria for pSS. While the European-American Consensus Group criteria^26^ include symptoms of dry mouth and dry eyes, the ACR-criteria include only objective criteria of reduced secretion of saliva and tears, ocular staining, positive focus score, and presence of anti-SSA/Ro in serum^35^. Both classification criteria for pSS require that the patients have anti-SSA/Ro antibodies and/or have a positive focus score, but do not include germinal centers. The epithelial gene expression profiles we describe show some association with clinical/pathological features of SS, but such molecular similarities are also evident in non-SS patients. This may be a consequence of the stochastic nature of clinical/pathological characteristics currently used to stratify patients into non-SS and SS groups.

Changes in SS epithelial and stromal tissue biology have been increasingly shown to play a role in SS pathobiology^13,36^. Previous studies revealed alterations in subpopulations of epithelial cells in NOD/ShiLtJ mouse model of SS, revealing a decrease in the tissue area of secretory cells in favor of ductal epithelial cells and expansion of keratin 5-positive basal cells^37^. Additional studies highlighted the key roles of the innate and adaptive immune systems, as well as stromal cells, in promoting the pro-inflammatory milieu^38,39^. A more recent report using an LCM approach coupled with RNAseq analyses described identification of cell-type and disease-specific markers in SS patients^40^. However, the latter study did not strictly focus on salivary gland epithelia, and thus precluded identification of gene signatures associated only with the epithelial compartment. Additionally, recent analyses of PBMCs from SS patients provided evidence for the involvement of the perturbed long non-conding RNAs (lncRNAs)—microRNAs axes in the expression of genes involved in the pathogenesis of SS, including those with roles in B cell function and extracellular matrix remodeling, among others^13^. Here, our results identifying IL-15 and BAFF cytokines with important functions in B cell physiology, as well as SPOCK2 proteoglycan contributing to the increasingly recognized roles of the stromal compartment in the pathogenesis of SS^36^, provide important new information into their epithelial origin in SS.

In addition to signals known to be enriched in SS, including IFN-γ and JAK/STAT-regulated genes, and genes encoding secreted factors implicated in immune responses, our studies identified LTF, also known as lactoferrin (LF), to be upregulated in expression in SS epithelia compared to non-SS tissues. Increased levels of salivary LTF with some relationship to the labial salivary gland histopathology grade were reported previously^28^, while no significant changes in LTF expression were detected in tear secretions in SS^41^. LTF is an iron-binding multifunctional glycoprotein known to function in the prevention of infections, late onset sepsis, and necrotizing enterocolitis^29^. LTF has been shown to stimulate a balanced T-helper-1/T-helper-2 cytokine immune response and to be a part of the innate defense, mainly at mucosa^42^. In our study, the observed increase in LTF gene expression in SS ductal epithelia suggests that it may be a relevant biomarker predictive of early disease with a potential causal effect on the induction of immune response.

Our data also show that MMP7 and BMP3 transcripts are upregulated in labial salivary glands from SS patients. MMP7 is a member of ubiquitous MMPs with important functions in tissue remodeling during development, but whose inappropriate expression has been linked to tissue destruction in autoimmune rheumatoid arthritis^43^ and with corneal melting in patients with primary SS^30^. This suggests that upregulation of MMP7 in labial tissues from SS patients is likely to contribute to salivary gland destruction. In contrast, relatively less is known about the role of BMP3 in pSS. Previous studies documented increased expression of BMP6 in salivary and lacrimal glands from human SS patients independent of autoantibodies^19^. To date, increased expression of BMP3 has been linked to immune infiltration in rheumatoid arthritis and inhibition of bone repair^31^. Thus, our RNAseq-based identification of BMP3 in salivary epithelia from SS patients may provide additional mechanistic insights into how inflammation induces BMP3 expression to drive epithelial dysfunction in pSS.

Accumulating evidence suggests a role for the epithelium in the etiology of SS. Impaired epithelial secretion has been reported to develop independently of lymphocytic infiltration and frequently, defective secretion precedes the immune response during SS onset^2^. Epithelial defects are often accompanied by pro-fibrotic changes in the stroma indicating remodeling of the extracellular matrix and fibroblast activation^36^. Here, our findings align alterations in gene expression signatures in the salivary epithelia with different degrees of compromised salivary and lacrimal glands’ function. Collectively, these studies offer a molecular explanation for exocrine tissue dysfunction as it progresses to SS and suggest that transcriptional signatures may serve as predictive biomarkers for early and advanced disease.

## Methods

### Patient selection and sample collection

The study protocol was approved by the Norwegian Regional Committee for Medical and Health Research Ethics of West (REK 2009/686) and South-East Norway (REK 2010/1292-1). The study was performed in compliance with the tenets of the Declaration of Helsinki. Prior to participation in the study, written informed consent was obtained from all participants. The data was de-identifed prior to analysis.

Labial salivary glands were obtained from a total of 27 patients with sicca symptoms. Based on the American-European Consensus Criteria^26^, these patients were sub-divided into 13 pSS (focus score (FS) 1–4) and 14 non-SS sicca controls (FS < 1). Inclusion criteria were: SS-patients fulfilling the AECG classification for pSS; non-SS controls were patients evaluated for SS, but not fulfilling the AECG classification. Importantly, none of the non-SS controls had a positive focus score or positive anti-SSA/Ro or anti-SSB/La autoantibodies. All participants provided informed consent prior to the initiation of this study. Clinical characteristics of patients whose minor salivary gland (SG) biopsies were analyzed for RNAseq are summarized in Table 1 (Bergen cohort) and for RNAscope analyses in Table 3 (Oslo cohort). These included assessment of objective tests of antinuclear antibodies (ANA, anti-SSA/Ro, anti-SSB/La), saliva secretion (unstimulated flow of whole saliva), tear secretion by Schirmer test, FS, and germinal center (GC)-like structures, as well as subjective symptoms noted by the patients (dry mouth and dry eyes).

### Histopathology

Paraffin-embedded, formalin-fixed human salivary gland tissue sections, 5-µm-thick, were stained with hematoxylin and eosin (H&E) and focal lymphocytic infiltration areas in H&E slides were captured and assessed using Aperio digital technology (Leica Biosystem). Minor SG FS of SS patients was assessed based on the currently accepted criteria^4^. The sections were evaluated by one oral pathologist (KS) in order to determine their FS. The tissue sections were further assessed for the presence of GC-like structures both on H&E sections and with an additional staining with CD21 (a marker of follicular dendritic cells) to improve the reliability of GC identification^44^. Fresh frozen biopsies from the Bergen cohort characterized in Table 1, were used for RNAseq analyses. Additional minor SG biopsies from another patient cohort (Oslo cohort, Table 3) were used for immunofluorescence labeling and RNAscope analyses.

### Laser capture microdissection (LCM) analysis

Fresh surgical specimens were preserved in the Tissue-TEK OCT compound and frozen in isopentane, followed by storage at − 80 °C. Tissue sections (5-μm-thick) were stained with an ethanol-based cresyl violet and eosin stain, followed by examination under the microscope and annotation of epithelium and inflammatory infiltrates on digital images. Acinar and ductal epithelium was subsequently captured by LCM using a Leica LMD6 microdissection system with regions containing inflammatory cells being excluded.

### RNAseq analyses

#### RNA extraction and RNA sequencing

RNA was isolated from laser capture microdissected labial SG epithelia. Approximately 30–100 ng of RNA was isolated from each patient sample in 4 μl with concentration of 7.5–25 ng/μl. Libraries for RNAseq were prepared using the Clontech Mammalian SMARTer Stranded Total RNASeq Kit Pico Input and then sequenced on an Illumina HiSeq 2500 instrument.

#### Processing RNA-sequencing data

Paired-end RNA-sequencing reads for each sample were first trimmed for Illumina TruSeq adapters (AGATCGGAAGAGCACACGTCTGAACT CCAGTCAC and AGATCGGAAGAGCGTCGTGTAGGGAAAGAGTGTAGATCGGAAGA GCGTCGTGTAGGGAAAGAGTGTAGATCTCGGTGGTCGCCGTATCATT) using Cutadapt tool^45^, setting the minimum read quality threshold to 20 for the start and end of the reads, and 15 for the minimum read length. Reads were then aligned to a reference hg19 genome using Tophat2^46^, with the number of allowed read mismatches, gap length and edit distance set to 1. Reads were then mapped to gene features using Ensembl hg19 (human genome release 19) genome annotations, and quantified as raw counts per feature using featureCount^47^ using default parameters.

#### RNA differential expression testing

Gene feature raw RNA expression counts were normalized and tested for differential expression between different sample groupings using DESeq2^48^. In our analyses, we did not include age as a variable to be adjusted for when performing differential expression testing, although we are aware that age can be a confounding factor. Pairwise comparisons for the non-SS and SS RNAseq data provided sufficient coverage to estimate the differential expression of moderately to highly expressed genes.

#### Gene set enrichment analysis

For testing enrichment of pathways, genes were first ranked (genome-wide) based on their signed average log-fold change between group comparisons (e.g. Sjogren’s vs control), using the pre-ranked GSEA tool v2.1.1 given a specific differential expression ranked list (based on the ‘t-statistic’ of differential expression). Data were then tested for enrichment of annotated hallmark gene sets from the molecular signature database (MSigDB)^49^ using the pre-ranked Gene Set Enrichment Analysis (GSEA) tool^50^. Pathway-based enrichment testing allowed the identification of coordinated sets of genes, and GSEA computationally determined if a defined set of genes was of statistical significance. We note that we did not use any fold-change cut-off here in addition to an FDR threshold.

### RNAscope analyses

Formalin-fixed, paraffin-embedded non-SS and SS patient minor SG biopsies from the Bergen and Oslo patient cohorts were cut into 4-μm-thick sections and mounted onto Superfrost Plus slides. Samples were deparafinized and in situ hybridization was conducted using the RNAscope 2.5 HD Assay Reagent Kit (ACDBio), probing for lactotransferrin Hs-LTF (LTF) RNA, matrix metalloproteinase 7 Hs-MMP7 (MMP7) RNA, and bone morphogenetic protein 3 (BMP3) RNA, Positive Control Probe Hs-PPIB, and Negative Control probe Dap-B according to the manufacturer’s instructions (ACDBio). After the final amplification, fast-red chromogenic detection was performed. The epithelial localizations of LTF, MMP7, and BMP3 were determined by counterstaining the tissue sections with pan-cytokeratin protein. Sections were blocked with 5% donkey serum-TBST (DS-TBST) and incubated in primary antibody 1:100 in 5% DS-TBST for 60 min, followed by incubation in 1:500 secondary goat anti-mouse conjugated with Alexa 647, counterstained with DAPI, and mounted in ProLong Gold Antifade and coverslipped. Image processing of the LTF/BMP3/MMP7 and pan-cytokeratin (pCK) signals was carried out on the free, open-source software CellProlifer v4.0.6 across all 2048X2048px images^32–34^ (http://www.cellprofiler.org). We utilized the ability to mask nuclei and epithelial cells, followed by merging the two masks to define a full epithelial cell. Nuclei outside the epithelial mask was discarded from further analysis. Following the estimation of epithelial cells, we measured the area of pixels of RNAscope probes within the of area covered by the epithelial mask to derive the percentage of area covered by a probe signal over all the epithelial areas (Supplementary Figure 1). This analysis was carried out on all patients on a minimum of 3 images per patient.

### Statistical analysis

All analyses were performed with GraphPad Prism statistical software version 8.4.3 (GraphPad Software Inc., La Jolla, CA, USA). Unpaired *t* tests were performed to compare values among groups for RNAscope of LTF, BMP3, and MMP7 expression. Each data point represents a patient. In order to determine the impact of age on gene expression, simple linear regression was calculated for each condition (Non-SS and SS), where age was the independent variable and expression of genes (LTF, BMP3, MMP7) quantified in the RNAscope experiment was the dependent variable. p < 0.05 considered to be statistically significant.

## Supplementary Information

Supplementary Figure 1.

Supplementary Table 1.
