# Supplementary material for: Gene expression alterations in salivary gland epithelia of Sjögren’s syndrome patients are associated with clinical and histopathological manifestations
Source: Sci Rep. 2021 May 27;11:11154. doi: 10.1038/s41598-021-90569-w (PMC8159963; doi:10.1038/s41598-021-90569-w)
Supplement: Supplementary file 1 — Supplementary Figure 1. [file 41598_2021_90569_MOESM1_ESM.pdf]

Supplementary Information

Gene expression alterations in salivary gland epithelia of Sjögren's Syndrome patients are associated with clinical and histopathological manifestations

Ariana Dela Cruz[1#], Vinay Kartha[2#], Andrew Tilston-Lunel[3#], Rongjuan Mi[1,3], Taylor L. Reynolds[4], Michael Mingueneau[4], Stefano Monti[2], Janicke L. Jensen[5], Kathrine Skarstein[6], Xaralabos Varelas[3*] and Maria A. Kukuruzinska[1*]

[1]Department of Translational Dental Medicine, Boston University School of Dental Medicine; [2]Department of Medicine, Boston University School of Medicine; [3]Department of Biochemistry, Boston University School of Medicine; [4]Immunology Research, Biogen Idec, Cambridge, Massachusetts; [5]Faculty of Dentistry, University of Oslo, Oslo, Norway; [6]Department of Clinical Medicine, University of Bergen, Bergen, Norway.

[#] These authors made equal contributions

* Correspondence and requests for materials should be addressed to either XV (xvarelas@bu.edu) or MAK (mkukuruz@bu.edu)

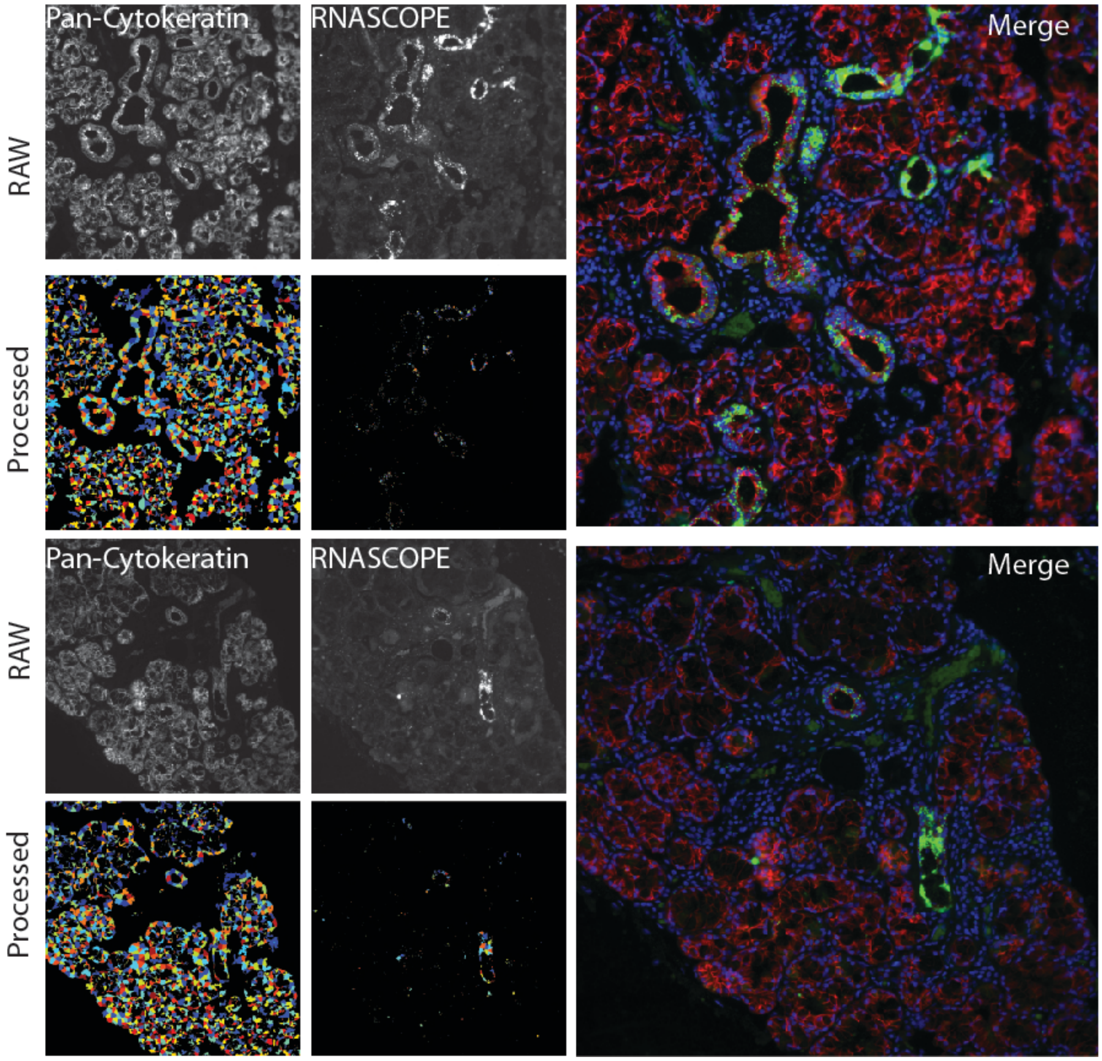

Figure S1. RNAscope image processing. Demonstration of image processing of the LTF/MMP7/BMP3 (RNASCOPE, green) and pan-cytokeratin signals (Pan-Cytokeratin, red) was carried out on CellProlifer 4.0.6. Nuclei (blue) and epithelial cells (red) were masked, followed by merging the two masks to define a full epithelial cell (Merge) using the CellProfiler image analysis software (www.cellprofiler.org). Nuclei outside the epithelial mask was discarded from further analysis (Processed, Pan-Cytokeratin). Following the estimation of epithelial cells, the area of pixels of RNAscope probes within the of area covered by the epithelial mask was measured (Processed, RNASCOPE) to derive the percentage of area covered by a probe signal over all the epithelial areas.

Supplementary Table

Table S1. Table of GSEA results with significantly up- or down-regulated Hallmark gene sets from laser-capture microdissected epithelium of SS vs Non-SS labial salivary glands. Columns: NES: GSEA normalized enrichment score of gene set, NOM p-val: nominal p-value, FDR q-val: false discovery rate, FWER p-val: family-wise error rate, gene names: list of genes differentially expressed in each gene set.
